# Interspecies metabolite transfer fuels the methionine metabolism of *Fusobacterium nucleatum* to stimulate volatile methyl mercaptan production

**DOI:** 10.1128/msystems.00764-23

**Published:** 2024-01-30

**Authors:** Takeshi Hara, Akito Sakanaka, Richard J. Lamont, Atsuo Amano, Masae Kuboniwa

**Affiliations:** 1 Graduate School of Pharmaceutical Sciences, Osaka University, Osaka, Japan; 2 Advanced Technology Institute, Mandom Corporation, Osaka, Japan; 3 Department of Preventive Density, Osaka University Graduate School of Dentistry, Suita, Osaka, Japan; 4 Department of Oral Immunology and Infectious Diseases, School of Dentistry, University of Louisville, Louisville, Kentucky, USA; APC Microbiome Ireland, Cork, Ireland

**Keywords:** metabolic interaction, methyl mercaptan, *Fusobacterium nucleatum*, *Streptococcus gordonii*, polyamines, methionine pathway, halitosis, one-carbon pool

## Abstract

The major oral odor compound methyl mercaptan (CH_3_SH) is strongly associated with halitosis and periodontitis. CH_3_SH production stems from the metabolism of polymicrobial communities in periodontal pockets and on the tongue dorsum. However, understanding of CH_3_SH-producing oral bacteria and their interactions is limited. This study aimed to investigate CH_3_SH production by major oral bacteria and the impact of interspecies interactions on its generation. Using a newly constructed large-volume anaerobic noncontact coculture system, *Fusobacterium nucleatum* was found to be a potent producer of CH_3_SH, with that production stimulated by metabolic interactions with *Streptococcus gordonii*, an early dental plaque colonizer. Furthermore, analysis of extracellular amino acids using an *S. gordonii* arginine-ornithine antiporter (ArcD) mutant demonstrated that ornithine excreted from *S. gordonii* is a key contributor to increased CH_3_SH production by *F. nucleatum*. Further study with ^13^C, ^15^N-methionine, as well as gene expression analysis, revealed that ornithine secreted by *S. gordonii* increased the demand for methionine through accelerated polyamine synthesis by *F. nucleatum*, leading to elevated methionine pathway activity and CH_3_SH production. Collectively, these findings suggest that interaction between *S. gordonii* and *F. nucleatum* plays a key role in CH_3_SH production, providing a new insight into the mechanism of CH_3_SH generation in oral microbial communities. A better understanding of the underlying interactions among oral bacteria involved in CH_3_SH generation can lead to the development of more appropriate prophylactic approaches to treat halitosis and periodontitis. An intervention approach like selectively disrupting this interspecies network could also offer a powerful therapeutic strategy.

**IMPORTANCE:**

Halitosis can have a significant impact on the social life of affected individuals. Among oral odor compounds, CH_3_SH has a low olfactory threshold and halitosis is a result of its production. Recently, there has been a growing interest in the collective properties of oral polymicrobial communities, regarded as important for the development of oral diseases, which are shaped by physical and metabolic interactions among community participants. However, it has yet to be investigated whether interspecies interactions have an impact on the production of volatile compounds, leading to the development of halitosis. The present findings provide mechanistic insights indicating that ornithine, a metabolite excreted by *Streptococcus gordonii*, promotes polyamine synthesis by *Fusobacterium nucleatum*, resulting in a compensatory increase in demand for methionine, which results in elevated methionine pathway activity and CH_3_SH production. Elucidation of the mechanisms related to CH_3_SH production is expected to lead to the development of new strategies for managing halitosis.

## INTRODUCTION

Bad breath, commonly referred to as halitosis, can have a significant negative impact on the social life of affected individuals. Sources of oral odor can be generally divided into microbial communities in periodontal pockets and those on the tongue dorsal surface, from which volatile compounds are emitted due to the bacterial metabolism of odorless proteins, peptides, blood, gingival crevicular fluid, and food retained on oral surfaces. Volatile sulfur compounds (VSCs), polyamines, short-chain fatty acids, and indoles are known odor-causing compounds emanating from the oral cavity (1–3). Among those, VSCs, composed of hydrogen sulfide (H_2_S), methyl mercaptan (CH_3_SH), and dimethyl sulfide [(CH_3_)_2_S], are strongly associated with oral odor (4, 5). In particular, CH_3_SH has an extremely low olfactory threshold value of 0.070 ppb, as compared to 0.41 ppb for H_2_S and 3.0 ppb for (CH_3_)_2_S (6), indicating that even a small amount causes an unpleasant odor. In addition, CH_3_SH production is associated with periodontal disease and is used as a biomarker of periodontitis (7, 8). Previous studies have also shown that CH_3_SH exhibits high toxicity toward periodontal tissues at a low concentration by inhibiting the synthesis of proteins, collagens, and DNA in human gingival fibroblasts, as well as by increasing the membrane permeability of oral mucosal epithelium (9–12). Together, these findings suggest that CH_3_SH is not only a key odor compound for halitosis but also a possible contributor to the pathogenesis of periodontitis.

CH_3_SH is produced by bacterial degradation of l-methionine by l-methionine-α-deamino-γ-mercaptomethane-lyase (METase) (13). Previous research has found that periodontal bacteria, such as the Gram-negative anaerobes *Fusobacterium nucleatum*, *Prevotella intermedia*, and *Porphyromonas gingivalis*, contribute to CH_3_SH production (13, 14). However, knowledge regarding CH_3_SH-producing oral bacteria is largely based on the findings from enzyme assays, in which substrates are reacted with enzymes extracted from oral bacteria (15, 16). Moreover, most related studies that used bacterial cultures employed a relatively small-volume incubation system, such as 3.5 mL vials with gas-tight valves (17, 18). Quantifying CH_3_SH, heavier than air and prone to settling at the bottom of vials, can cause large variations in its amounts and difficulty with the detection of a trace quantity. Also, in experiments that require a substantial bacterial amount, such as gene expression analysis, small-volume incubation systems are not suitable.

In recent years, there has been growing interest in the collective properties of oral polymicrobial communities, regarded as an important factor for the development of oral diseases such as periodontitis and dental caries (19, 20). These properties may be shaped by physical and chemical interactions among community participants including streptococci and actinomycetes, which are nonpathogenic commensals (21–24). In particular, a subset of oral streptococci has been shown to engage in interspecies communications with oral pathogens, such as the provision of attachment sites through coaggregation-mediated adhesion and exchange of diffusible signaling molecules termed autoinducers (AIs) via quorum sensing (QS) (25–33). Furthermore, studies recently conducted have reported that streptococcal metabolites, such as lactate, ornithine, and para-aminobenzoic acid, have a major impact on oral community properties, resulting in an increased risk of periodontitis (34–37). However, no known study has investigated whether interspecies communication by streptococci has an impact on CH_3_SH generation.

Using a newly constructed large-volume anaerobic noncontact coculture system, the present study was conducted to assess CH_3_SH production by major oral bacteria and the impact of interspecies interactions on that production. The findings show that *F. nucleatum* is a potent producer of CH_3_SH, with that production stimulated by metabolic interactions with *Streptococcus gordonii*. Additionally, this phenomenon was found to be driven by ornithine excreted from *S. gordonii*, which boosts the demand for methionine via increased synthesis of polyamines by *F. nucleatum*, resulting in elevated methionine metabolism as well as CH_3_SH production.

## MATERIALS AND METHODS

### Strains, media, and growth conditions

*Actinomyces naeslundii* ATCC 19039, *S. gordonii* DL1, and its isogenic Δ*arcD* mutant (37), *F. nucleatum* subsp. *nucleatum* ATCC 25586, *Filifactor alocis* ATCC 35846, *P. intermedia* ATCC 49046, and *P. gingivalis* ATCC 33277 were used as representative oral bacteria in this study. *S. gordonii* strains were precultured in an aerobic environment in Todd Hewitt broth at 37°C. *A. naeslundii*, *P. intermedia*, and *P. gingivalis* were anaerobically grown at 37°C in trypticase soy broth supplemented with 1.0 mg/mL yeast extract, 1.0 µg/mL menadione, and 5.0 µg/mL hemin. *F. nucleatum* was anaerobically grown at 37°C in medium containing 1.92 g/mL brain heart infusion broth, 1.0 g/mL trypticase peptone, 1 mg/mL yeast extract, 1.0 g/mL biosate peptone, 1.0 µg/mL menadione, 5.0 µg/mL hemin, 0.2 mM K_2_HPO_4_, 0.3 mM KH_2_PO_4_, 4.8 mM NaHCO_3_, 72 µM CaCl_2_, 1.4 mM NaCl, and 66 µM MgSO_4_. *F. alocis* was anaerobically grown in modified Gifu anaerobic medium (GAM) agar (Nissui Pharmaceutical, Tokyo, Japan). Precultures were performed in an anaerobic chamber (Concept Plus; Ruskinn Technology, Bridgend, UK) with an atmosphere containing 85% N_2_, 10% H_2_, and 5% CO_2_. When necessary, the antibiotic erythromycin (5 g/mL) was used as a selective marker for *S. gordonii*. The modified chemically defined medium (mCDM) used consisted of 58 mM K_2_HPO_4_, 15 mM KH_2_PO_4_, 10 mM (NH_4_) _2_SO_4_, 35 mM NaCl, 0.1 mM MnCl_2_•4H_2_O, 2 mM MgSO_4_•7H_2_O, 0.04 mM nicotinic acid, 0.1 mM pyridoxine-HCl, 0.01 mM pantothenic acid, 1.0 µM riboflavin, 0.3 µM thiamin-HCl, 0.05 µM D-biotin, 50 mM α-ketoglutaric acid, 5.6 mM D(+)-glucose, 4.0 mM l-glutamic acid, 1.0 mM l-arginine-HCl, and 0.1 mM l-tryptophan (38).

### Pretreatment

*A. naeslundii* (OD 1.4), *S. gordonii* (OD 1.4), *F. nucleatum* (OD 1.4), *P. intermedia* (OD 1.2), *F. alocis* (OD 1.2), and *P. gingivalis* (OD 1.3) were harvested by centrifugation (8,000 × *g* for 7 min at 4°C) and washed twice in phosphate buffer saline (PBS). Cells were adjusted at an OD_600_ of 1.0 in mCDM, supplemented with 0.5 to 5.0 mM l-methionine, 5.0 mM l-cysteine, 5.0 mM dl-homocysteine, and 5.0 mM serine, as necessary (mCDM solution). The mCDM solution was adjusted at pH 6.5 unless experimental pH conditions are specified.

### *In vitro* assays for CH_3_SH generation

#### Monoculture

Ten milliliters of the pretreated bacterial suspensions described above were added to 30 mL of mCDM solution.

#### Coculture

Using pretreated bacteria, 10 mL of *A. naeslundii* or *S. gordonii* along with 10 mL of *F. nucleatum*, *P. intermedia*, *F. alocis*, or *P. gingivalis* was mixed with 20 mL of mCDM solution.

#### Noncontact culture

For the cocultivation of two species of bacteria under a contactless condition, dialysis tubing (Spectra/Por 7 Dialysis Membrane Pretreated RC Tubing MWCO 1 kDa; Spectrum Laboratories, Inc., CA, USA) was used. The tubing was rinsed twice with sterile water and then autoclaved at 120°C for 15 min in distilled water. *F. nucleatum* with *S. gordonii* wild type (WT) or Δ*arcD* mutant at the late-exponential phase (1.0 to 1.5 OD units/mL) was adjusted to an OD_600_ of 1.0 in mCDM solution. The tubing was filled with 10 mL of *S. gordonii* WT or Δ*arcD* mutant and transferred to a flask, and then 10 mL of *F. nucleatum* and 20 mL of mCDM solution were added to the flask. For monocultures of *F. nucleatum*, after filling the tubing with 10 mL of mCDM solution, 10 mL of *F. nucleatum* and 20 mL of mCDM solution were added to the flask. For monocultures of *S. gordonii* WT and Δ*arcD* mutant, the tubing was filled with 10 mL of *S. gordonii* WT or Δ*arcD* mutant, and then 20 mL of mCDM solution was added to the flask.

All samples were incubated either anaerobically or microaerobically at 37°C for 16 h using a contact or noncontact type of culture system (Fig. S1). A set of four flasks of bacterial cultures for each experimental group were incubated in a jar (The GasPak 150 jar, Becton, Dickinson and Company, NJ, USA) at 37°C either anaerobically or microaerobically to minimize contamination by volatile compounds emitted from different experimental groups. Where required, anaerobic or microaerophilic atmospheric conditions were created by using the AnaeroPack gas generator (Mitsubishi Gas Chemical, Tokyo, Japan). Each flask removed from the jar was immediately covered with Parafilm (Parafilm M) after removing the rubber stopper to maintain anaerobic conditions. Additionally, a gas-tight syringe was inserted through the Parafilm and 1 mL of headspace gas was directly collected. The gas was quantitated by gas chromatography (GC; Shimadzu, Kyoto, Japan). When necessary, the gas was diluted to a ≥5 times volume with air. The gas was injected into the GC port of a GC-14B instrument equipped with a flame photometric detector (Shimadzu). A ZO-1H column (3.1 m × 3.2 mm i.d.; Shinwa Chemical Industries, Kyoto, Japan) was used. Nitrogen was utilized as the carrier gas at a constant flow rate of 50 mL/min. The oven and detector temperatures were kept at 70°C and 180°C, respectively. Identification of volatiles was based on matching retention time with those of authentic standards available. The three standard gases H_2_S, CH_3_SH, and (CH_3_)_2_S were produced in a permeation tube using a permeater (PD-1B; Gastec Corp., Tokyo, Japan) and collected into sampling bags. Each volatile compound was determined using calibration curves. Changes in bacterial density after cultivation were also measured at OD_600_.

### Analysis of extracellular metabolites

The time-course changes of extracellular metabolite compositions were investigated using *F. nucleatum*, *S. gordonii* WT, Δ*arcD* mutant, and their cocultures. For the monocultures, 3 mL of each bacterium at an OD_600_ of 1.0 in mCDM solution was mixed with 9 mL of mCDM solution in a six-well tissue culture plate. For the cocultures, 3 mL of *F. nucleatum* and *S. gordonii* WT or Δ*arcD* mutant at an OD_600_ of 1.0 in mCDM solution were mixed with 6 mL of mCDM solution in a six-well tissue culture plate. All sample solutions were anaerobically cultured at 37°C for 0, 6, or 12 h. The samples were passed through a 0.22-mm membrane filter (Millex-GP: Millipore, MA, USA) to remove bacteria and the supernatants were analyzed for amino acid concentrations using the Waters AccQ Amino Acid Analysis method with an ultra-performance liquid chromatography (UPLC) system (Waters ACQUITY H-Class; Waters, Milford, USA), consisting of a photodiode array (PDA) detector, column heater, sample manager, and binary solvent delivery system (supplemental experimental procedures).

### Metabolic flux analysis

Intracellular metabolic flux analyses were performed to investigate the fate of methionine incorporated by *F. nucleatum* when cocultured with *S. gordonii* WT. Cocultures of *F. nucleatum* with *S. gordonii* WT were performed in six-well Corning Costar Transwell plates (pore size 0.4 µm, 24 mm in diameter; Corning, NY, USA). *F. nucleatum* cells in the outer chamber were collected after incubation. [^13^C_5_, ^15^N] l-methionine (^13^C_5_, ^15^N, 98%; Taiyo Nippon Sanso Corp., Tokyo, Japan) was added at a final concentration of 10 mM to mCDM, with the following modified amino acid concentrations: 40 mM l-glutamic acid, 10 mM l-arginine-HCl, and 1.0 mM l-tryptophan. At the mid-exponential growth phase (0.5 to 1.0 OD units/mL), bacterial cells were harvested by centrifugation (7,670 × *g* for 7 min at 4°C), washed twice with PBS, and finally resuspended at 20 OD units/mL in mCDM containing 10 mM [^13^C_5_, ^15^N] l-methionine. *F. nucleatum* and *S. gordonii* cells were then inoculated at a density of 1.5 × 10^10^ CFU/well into a Transwell outer chamber (2.6 cm^3^) and inner chamber (1.5 cm^3^), respectively. Thereafter, anaerobic incubation was performed at 37°C for 0, 1, 2, 3, or 6 h. *F. nucleatum* cells obtained at each time point were harvested by centrifugation (7670 × *g* for 7 min at 4°C) and intracellular metabolites were extracted with 100% methanol. The analyses were performed using capillary electrophoresis time-of-flight mass spectrometry (CE-TOF-MS), as described in supplemental experimental procedures.

### Ornithine supplementation and ornithine decarboxylase inhibitor

l-Ornithine was added at a final concentration of 0.1 to 1.0 mM to the mCDM solution containing 1.0 mM l-methionine, while *F. nucleatum* cells were incubated using the anaerobic system described above. The inhibitory effects of dl-α-difluoromethylornithine hydrochloride monohydrate (DFMO; Tokyo Kasei Kogyo, Tokyo, Japan) on CH_3_SH generation were evaluated using cocultures of *F. nucleatum* and *S. gordonii* at final concentrations of 0.01 to 1.0 mM.

### Quantitation of mRNA transcripts

Following incubation in the noncontact coculture system in mCDM (pH 6.5) containing 1.0 mM l-methionine for 8 or 16 h at 37°C, bacterial cells in 27 mL of each monocultured and cocultured bacterial solution were collected by centrifugation (8,000 × *g* for 7 min at 4°C). The cells were then resuspended in 3 mL of RNAprotect Bacteria Reagent (Qiagen, Hilden, Germany). After incubation for 5 min at room temperature, 1 mL of the bacterial solution was collected and immediately frozen, then treated with 20 µL of proteinase K (Qiagen) and 200 µL of 15 mg/mL lysozyme at 55°C, and subjected to centrifugation at 1,000 rpm for 15 min. RNA isolation was performed with 1 mL of TRIzol reagent (Life Technologies Corp., CA, USA) and an RNeasy kit (Qiagen). cDNA synthesis along with the removal of genomic DNA was performed using iScript master mix (Bio-Rad, CA, USA) according to the manufacturer’s instructions. Real-time PCR assays were performed with a KAPA SYBR Fast kit (KAPA Biosystems, MA, USA), following the supplied protocol. Designed primers are shown in Table S1. To determine gene expression, a comparative Ct method was used.

### Statistical analysis

All statistical analyses were performed using Excel (Office365) with the Statcel4 software package (OMS Publishing Inc., Saitama, Japan). Different statistical tests were used for different experiments, as indicated in the corresponding figure legends.

## RESULTS

### Enhancement of CH_3_SH generation in cocultures and noncontact cocultures

**Fig 1:**
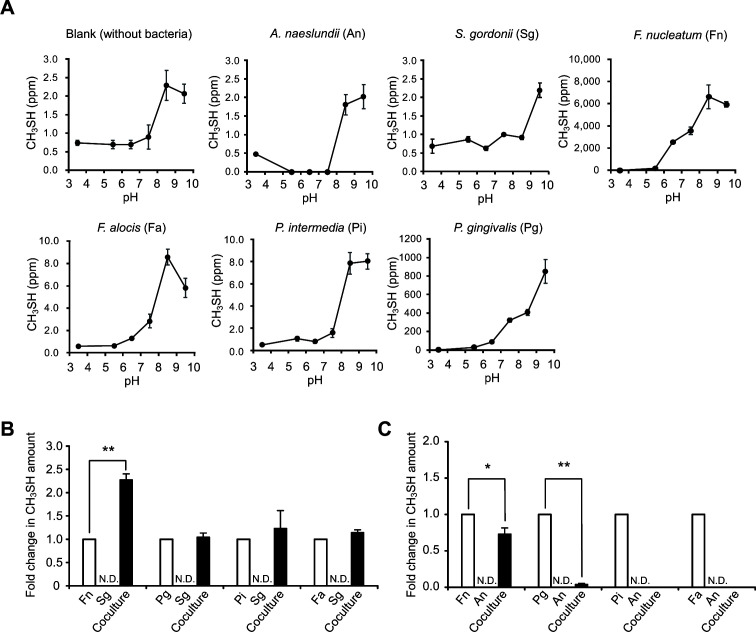
CH_3_SH generation by l-methionine metabolism of oral bacteria. (**A**) Changes in CH_3_SH production under various pH conditions. Bacterial cultures were supplemented with 5.0 mM l-methionine. Results are normalized with the final OD and shown as the mean ± SD of three independent experiments. (**B, C**) Enhancement of CH_3_SH production by coculturing with *S. gordonii* (**B**) or *A. naeslundii* (**C**). Bacterial cultures were supplemented with 0.5 mM l-methionine and adjusted at the final pH 6.5. Results are shown as the mean ± SD of three independent experiments. *P < 0.05, **P < 0.01 (two-tailed paired *t*-test); N.D., not detected. Fold changes in CH_3_SH amount were calculated using the following equation: fold = (amount of CH_3_SH formation in coculture)/(amount of CH_3_SH formation in single culture of *F. nucleatum*, *P. gingivalis*, *P. intermedia,* or *F. alocis*) + (amount of CH_3_SH formation in single culture of *S. gordonii* or *A. naeslundii*). All cultures were anaerobically incubated for 16 h, as described in Materials and Methods.

To assess the ability of oral bacterial organisms to produce CH_3_SH under anaerobic culture conditions, *A. naeslundii* and *S. gordonii* (early colonizers), *F. nucleatum* and *P. intermedia* (mid colonizers), and *F. alocis* and *P. gingivalis* (late colonizers) were selected as representative oral bacteria. *F. nucleatum* exhibited the highest CH_3_SH production among the oral bacteria tested, with the largest amount released at pH 8.5 (Fig. 1A). The production of CH_3_SH by *P. gingivalis* increased gradually with increasing pH, but its maximum concentration was about one-eighth of the maximum concentration in *F. nucleatum*. In contrast, both *A. naeslundii* and *S. gordonii* when cultured alone produced negligible amounts of CH_3_SH (Table 1). Only the OD value of *A. naeslundii* increased after 16 h of incubation in mCDM supplemented with 5 mM l-methionine, while the values of the other bacteria were virtually unchanged (Table S2).

**TABLE 1:** Precursors for CH_3_SH production by oral bacteria^*a*^

	CH_3_SH production (ppm)			
Bacterial strain	Substrates			
	l-Cysteine	l-Methionine	dl-Homocysteine	l-Serine
No bacteria (blank)	N.D.	0.17 ± 0.04	N.D.	N.D.
*Streptococcus gordonii* DL1	N.D.	0.36 ± 0.11	N.D.	N.D.
*Actinomyces naeslundii* ATCC19039	N.D.	N.D.	N.D.	N.D.
*Fusobacterium nucleatum* subsp. nucleatum ATCC25586	N.D.	477.71 ± 26.30	N.D.	N.D.
*Prevotella intermedia* ATCC49046	N.D.	0.18 ± 0.01	N.D.	N.D.
*Filifactor alocis* ATCC35846	N.D.	0.80 ± 0.15	N.D.	N.D.
*Porphyromonas gingivalis* ATCC33277	N.D.	14.39 ± 2.93	0.44 ± 0.02	N.D.

^*a*^ Bacterial cultures were supplemented with 5 mM of each substrate and anaerobically incubated for 16 h. Blank samples were cultured without bacteria. Data are shown as the mean ± SD of three independent experiments. N.D., not detected.

**Fig 2:**
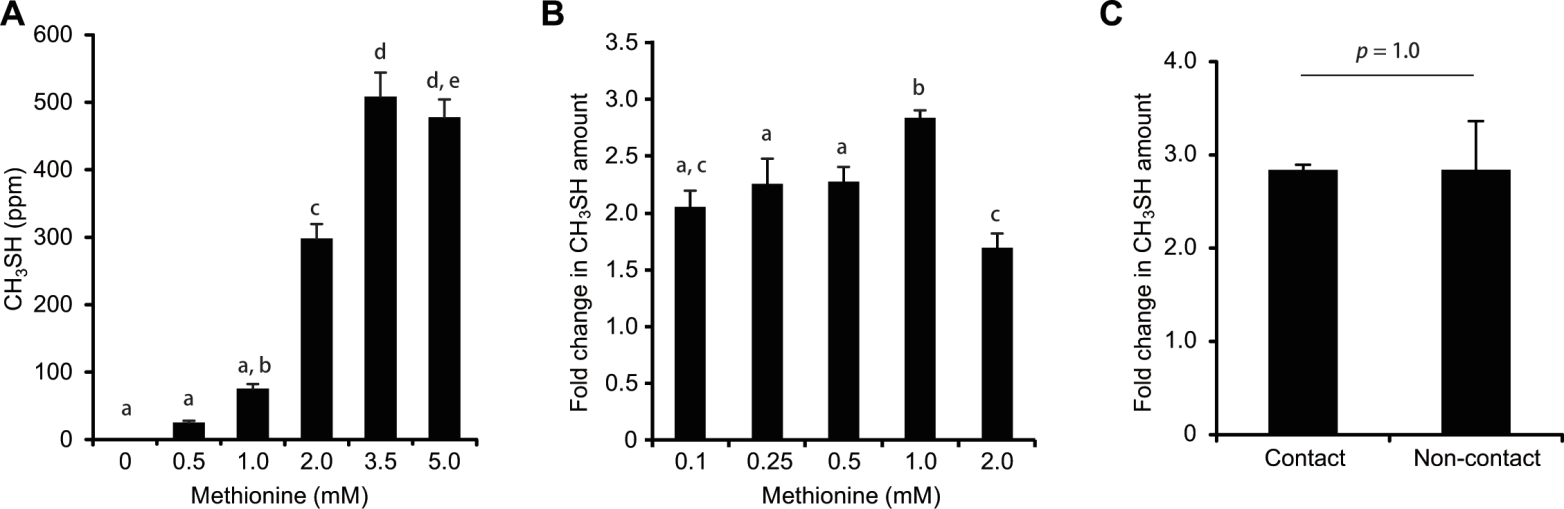
Changes in CH_3_SH production in *F. nucleatum* single cultures and cocultures with *S. gordonii*. (**A**) Relationships between the amount of CH_3_SH production and methionine concentration in mCDM in *F. nucleatum* single cultures. Data are shown as the mean ± SD of three independent experiments. In case of no significant difference between groups, the same alphabets are denoted (one-way ANOVA, followed by Tukey–Kramer post-hoc test, significance level; *P* < 0.01). (**B**) Fold changes in CH_3_SH level by the addition of various concentrations of methionine in cocultures of *F. nucleatum* and *S. gordonii*. Each fold change in CH_3_SH amount indicates multiples of CH_3_SH amount in cocultures of *F. nucleatum* and *S. gordonii* when the amount of CH_3_SH production in *F. nucleatum* monoculture under each condition is set as 1. Data are shown as the mean ± SD of three independent experiments. In case of no significant difference between groups, the same alphabets are denoted (one-way ANOVA, followed by Tukey–Kramer post-hoc test, significance level; *P* < 0.01). (**C**) Changes in CH_3_SH production in *F. nucleatum* and *S. gordonii* cocultures in contact or non-contact culture systems. The bacterial cultures were supplemented with 1.0 mM l-methionine. Each fold change in CH_3_SH amount indicates multiples of CH_3_SH amount in cocultures of *F. nucleatum* and *S. gordonii* when the amount of CH_3_SH production in *F. nucleatum* monoculture under each condition is set as 1. Results are shown as the mean ± SD of four independent experiments. Fold changes in CH_3_SH amount were calculated using the following equation: fold = (amount of CH_3_SH formation in coculture)/[(amount of CH_3_SH formation in single culture of *F. nucleatum*) + (amount of CH_3_SH formation in single culture of *S. gordonii*)]. All cultures were anaerobically incubated for 16 h, as described in Materials and Methods. A two-tailed *t*-test was performed to calculate the *P*-value.

Next, the effects of coculturing early colonizers with mid- or late colonizers on the enhancement of CH_3_SH generation were examined. When *F. nucleatum* was cocultured with *S. gordonii*, there was an approximately 2.3-fold increase in the amount of CH_3_SH production as compared to CH_3_SH levels in their respective monocultures (Fig. 1B). On the other hand, the addition of *A. naeslundii* significantly suppressed CH_3_SH generation in *F. nucleatum*, *P. gingivalis*, and *P. intermedia* cultures (Fig. 1C). *F. nucleatum* alone yielded the highest amount (approximately 500 ppm) of CH_3_SH with 3.5 to 5.0 mM l-methionine added at pH 6.5 (Fig. 2A), while the greatest level of enhancement (approximately 3-fold) in CH_3_SH production was observed in cocultures of *F. nucleatum* with *S. gordonii* when 1 mM l-methionine was added (Fig. 2B), indicating that *S. gordonii* can boost CH_3_SH production by *F. nucleatum* with lower concentrations of methionine.

To determine whether physical interactions between *F. nucleatum* and *S. gordonii* contribute to enhanced production of CH_3_SH, these species were cocultured without contact, and the findings were assessed (Fig. S1). The presence of *S. gordonii* in both contact and noncontact cocultures was found to increase CH_3_SH levels by up to 3-fold as compared to CH_3_SH levels in respective monocultures (Fig. 2C). These findings indicate that enhancement of CH_3_SH production by cocultured *F. nucleatum* and *S. gordonii* is due to an exchange of diffusible factors rather than through physical contact.

Furthermore, the effect of 6% oxygen concentration in the culture environment on CH_3_SH production was confirmed. Although there was no difference in CH_3_SH production in *F. nucleatum* monocultures, there was a significant decrease in the production of CH_3_SH when *F. nucleatum* and *S. gordonii* were cocultured in a microaerophilic environment than in an anaerobic environment (*P* < 0.01, Fig. S2).

### Noninvolvement of AI-2-based QS system in CH_3_SH production

To examine the involvement of an AI-2-based QS system, enhancement of CH_3_SH production was assessed by the addition of 4, 5-dihydroxy-2, 3-pentanedione (DPD), an AI-2 precursor, from which the LuxS enzyme catalyzes conversion in *F. nucleatum* (39). The results showed that DPD/AI-2 had no significant effect on CH_3_SH production as compared to the control samples (Fig. S3), indicating that an AI-2-based QS system is not involved in increased CH_3_SH production.

### Contribution of ornithine to increased CH_3_SH production when cocultured with *S. gordonii*

**Fig 3:**
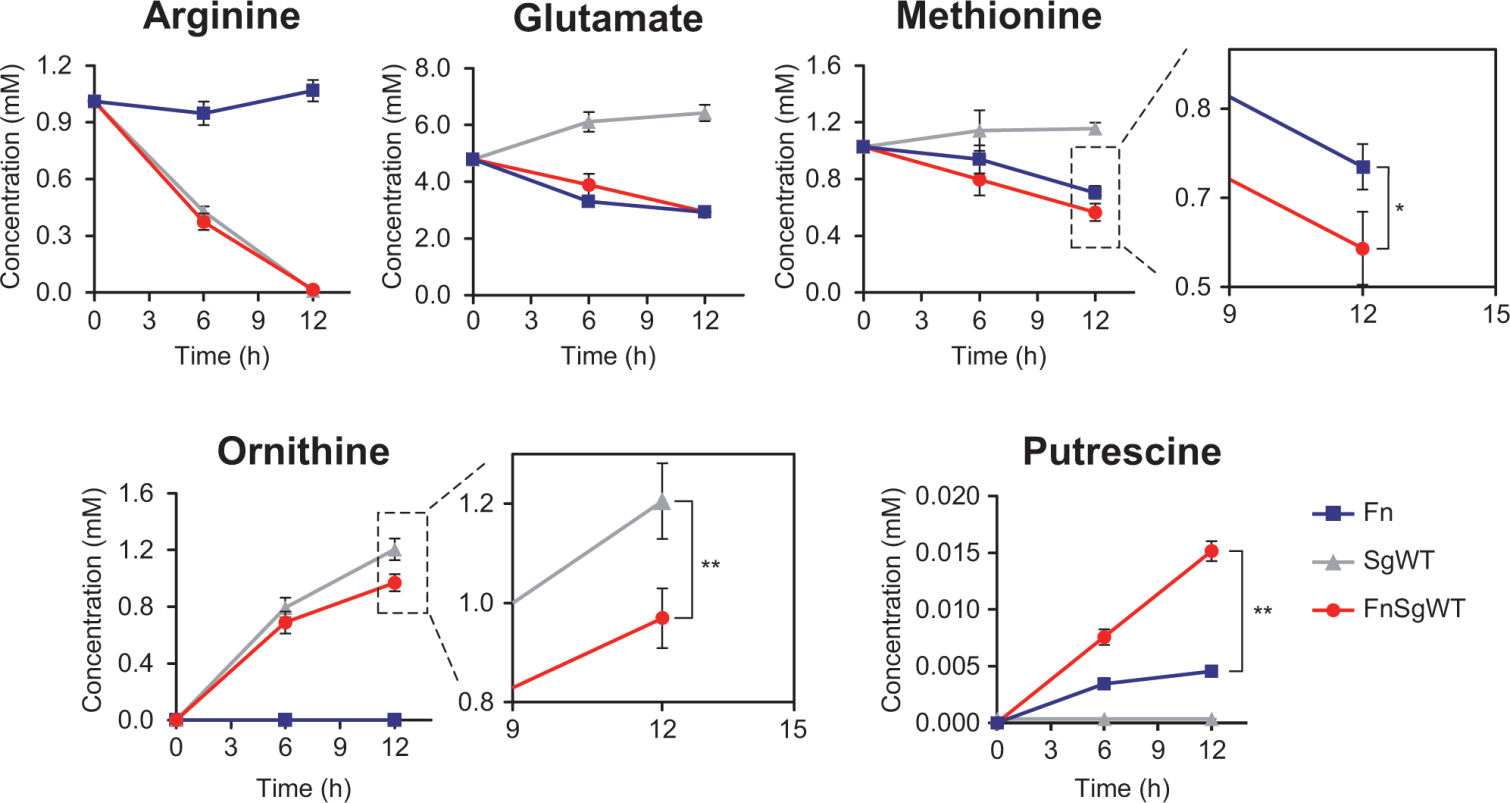
Time course of changes in extracellular metabolites in culture fluids. Lines indicate *F. nucleatum* (blue) and *S. gordonii* WT (gray) monocultures and *F. nucleatum* and *S. gordonii* WT (red) coculture. Results are shown as the mean ± SD of three independent experiments. *P < 0.05, **P < 0.01 (one-way ANOVA, followed by Tukey–Kramer post-hoc test) for methionine and ornithine metabolism.

To examine whether metabolic interactions underlie the promotion of CH_3_SH generation associated with coculturing, time-course changes of extracellular metabolites in cocultures of *F. nucleatum* and *S. gordonii* WT and also monocultures of each strain were assessed using UPLC (Fig. 3). *F. nucleatum* gradually consumed methionine, about 50% in the substrate present during 12 h of incubation, while *S. gordonii* showed a low level of consumption. On the other hand, cocultures of *F. nucleatum* and *S. gordonii* WT exhibited the highest level of consumption of methionine (*P* < 0.05, vs. *F. nucleatum* monocultures). Furthermore, monocultures of *S. gordonii* WT and cocultures with *F. nucleatum* showed depleted arginine, as well as release of 1.2 and 0.95 mM ornithine, respectively (*P* < 0.01), suggesting an uptake of 0.25 mM ornithine by *F. nucleatum* under coculture conditions. Putrescine levels in cocultures of *F. nucleatum* with *S. gordonii* WT were also markedly increased, indicating that putrescine excretion was accelerated by *S. gordonii*. Glutamate present in the mCDM was found to be gradually taken up by *F. nucleatum* but not *S. gordonii*.

**Fig 4:**
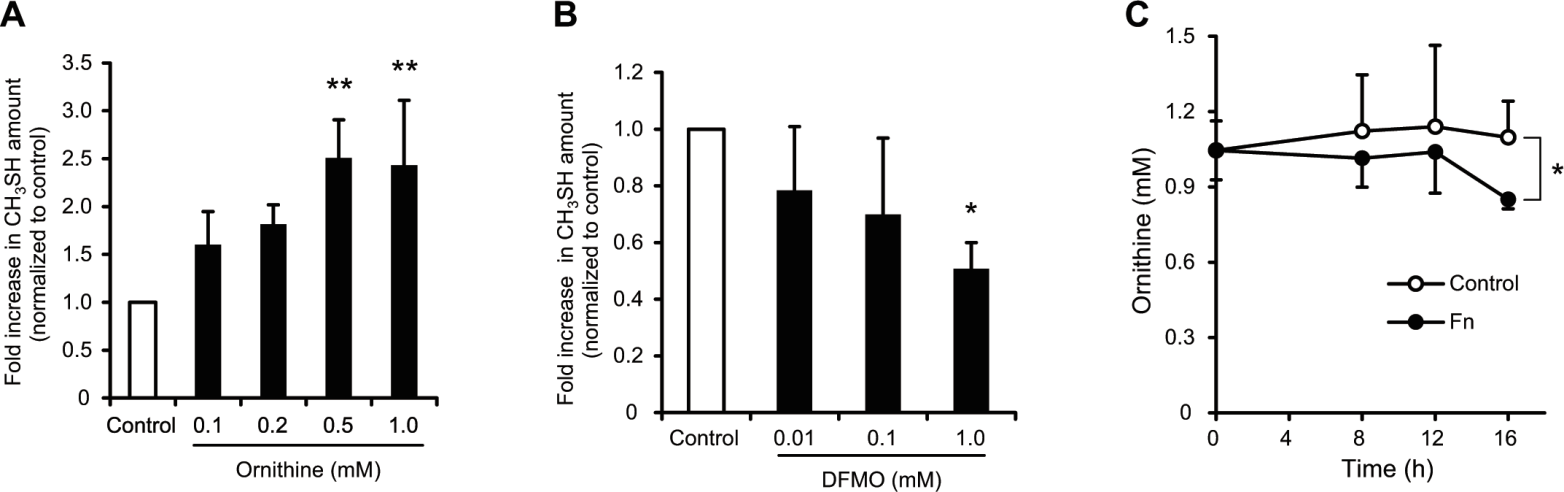
Effects of ornithine on enhancement of CH_3_SH generation. (**A**) Fold changes in CH_3_SH level by the addition of various concentrations of ornithine to *F. nucleatum* monocultures. The fold increase was normalized to that of the control sample without ornithine. *F. nucleatum* cultures were supplemented with 1.0 mM l-methionine and ornithine, then anaerobically incubated for 16 h. Results are shown as the mean ± SD of three independent experiments. **P < 0.01 (one-way ANOVA, followed by Dunnett’s test). (**B**) Inhibition of CH_3_SH generation by DFMO. The fold increase was normalized to that of the control sample without DFMO. Mixtures of *F. nucleatum* and *S. gordonii* cocultures supplemented with 1.0 mM l-methionine were anaerobically incubated for 16 h. Results are shown as the mean ± SD of three independent experiments. *P < 0.05 (one-way ANOVA, followed by Dunnett’s test). (**C**) Uptake of ornithine by *F. nucleatum*. Control indicates a sample without *F. nucleatum*. Results are shown as the mean ± SD of three independent experiments. *P < 0.05 for indicated time point (two-tailed *t*-test).

Next, we determined whether the addition of ornithine increased CH_3_SH production by *F. nucleatum*. Ornithine added at 0.1 or 0.5 mM provided a significant increase in the production of CH_3_SH (2.5-fold as compared to that without ornithine) (Fig. 4A), while bacterial growth was not significantly affected (data not shown). In addition, DFMO, an inhibitor of ornithine decarboxylase (ODC) (40, 41), diminished *S. gordonii*-induced stimulation of CH_3_SH generation in cocultures of *F. nucleatum* and *S. gordonii*, in a dose-response manner, with 1.0 mM significantly halving the amount produced (Fig. 4B). Moreover, extracellular ornithine was taken up by *F. nucleatum* over time (Fig. 4C). Together, these results suggest that ornithine metabolism by ODC of *F. nucleatum* enhances CH_3_SH production in the presence of ornithine.

### Cocultures of *F. nucleatum* and *S. gordonii* Δ*arcD*

ArcD of *S. gordonii* has been shown to mediate arginine uptake and concomitant ornithine export (42–44). To confirm whether ornithine from *S. gordonii* causes increased production of CH_3_SH by *F. nucleatum*, an *arcD*-deletion mutant strain was generated and CH_3_SH production in cocultures with *F. nucleatum* was evaluated. The presence of the Δ*arcD* mutant in both contact and noncontact cultures failed to increase CH_3_SH production by *F. nucleatum* (Fig. S4). Additionally, the Δ*arcD* mutant exhibited reduced levels of arginine uptake and ornithine export (Fig. S5). These results indicate that ornithine from *S. gordonii* is a key metabolite for enhancing CH_3_SH generation by *F. nucleatum*. Cocultures with Δ*arcD* mutants also showed diminished methionine utilization by *F. nucleatum* as compared to the WT strain (*P* < 0.01) (Fig. S5), whereas no significant difference was found between the amounts of methionine consumed by *F. nucleatum* in monocultures and cocultures with the Δ*arcD* mutant (Fig. S5). It is thus considered that the uptake of methionine by *F. nucleatum* is promoted by ornithine from *S. gordonii*, leading to enhanced CH_3_SH generation.

### Methionine metabolism of *F. nucleatum* under cocultivation condition

**Fig 5:**
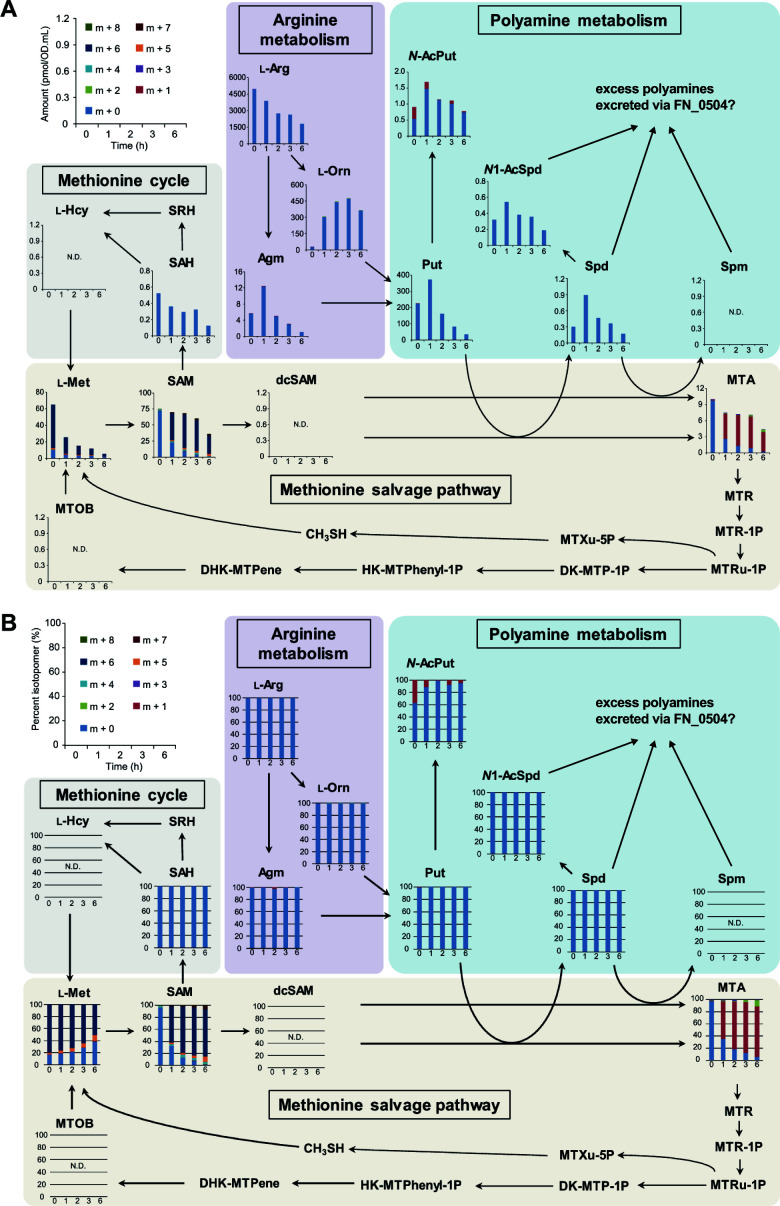
Flux profiling of ^13^C, ^15^N-labeled methionine salvage pathway metabolites. Quantitation values for (**A**) isotopomers and (**B**) the ratio of each as compared to total metabolites of methionine cycle and methionine salvage pathway metabolites from [^13^C_5_, ^15^N] l-methionine in *F. nucleatum*. Bacterial cultures were supplemented with 10 mM [^13^C_5_, ^15^N] l-methionine and anaerobically incubated for 6 h. Values obtained after omitting the abundance of naturally occurring isotopes for each detected metabolite were used. Results are shown as mean values from three independent experiments. l-Met, l-methionine; SAM, *S*-adenosyl- l-methionine; SAH, *S*-adenosyl- l-homocysteine; SRH, *S*-ribosyl-l-homocysteine; l-Hcy, l-homocysteine; dcSAM, *S*-adenosylmethioninamine; MTA, 5'-methylthioadenosine; l-Arg, l-arginine; Agm, agmatine; l-Orn; l-ornithine; Put, putrescine; *N*-AcPut, *N*-acetylputrescine; *N*1-AcSpd, *N*1-acetylspermidine; Spd, spermidine; Spm, spermine; MTR, 5-methylthio-d-ribose; MTR-1P, *S*-methyl-5-thio-d-ribose 1-phosphate; MTRu-1P, *S*-methyl-5-thio-d-ribulose 1-phosphate; MTXu-5P, 1-(methylthio)xylulose 5-phosphate; CH_3_SH, methyl mercaptan; DK-MTP-1P, 2,3-diketo-5-methyl-thiopentyl-1-phosphate; HK-MTPhenyl-1P, 2-hydroxy-3-keto-5-methylthiopentenyl-1-phosphate; DHK-MTPene, 1,2-dihydroxy-5-(methylthio) Pent-1-en-3-one; MTOB, 4-methylthio-2-oxobutanoic acid; N.D., not detected.

Using ^13^C/^15^N-labeled methionine, the fate of methionine in *F. nucleatum* cells was examined to elucidate the intracellular metabolic dynamics underlying enhanced CH_3_SH production in the presence of *S. gordonii*. Eleven of the 17 targeted metabolites were detected, and changes in 19 different ^13^C, ^15^N isotopomers related to methionine metabolism were determined using CE-TOF-MS (Fig. S6). The findings showed that fully labeled methionine (m + 6) was instantly incorporated into *F. nucleatum* cells and then markedly decreased over time (Fig. 5A). Furthermore, intracellular accumulation of labeled S-adenosyl-l-methionine (SAM; m + 6) derived from fully labeled methionine as well as labeled S-adenosylmethioninamine (MTA; m + 1) derived from SAM (m + 6) was also noted and then they were gradually consumed (Fig. 5A). The increase in intracellular level of ornithine reached a peak at 3 h, while the increased levels of polyamines, including putrescine, spermidine, and their acetyl derivatives, peaked at 1 h, after which they were consumed (Fig. 5A). Although intracellular S-adenosyl-L-homocysteine (SAH) levels were gradually decreased, with labeled SAH (m + 5) undetected, the ratio of labeled methionine (m + 5) showed an increase over time, indicative of methionine regeneration from L-homocysteine via SAH (methionine cycle) (Fig. 5B). Collectively, these results suggest that methionine mainly enters the methionine cycle and polyamine biosynthesis pathway in *F. nucleatum* cells when cocultured with *S. gordonii*.

### Gene expression in *F. nucleatum* in cocultures with *S. gordonii*

**Fig 6:**
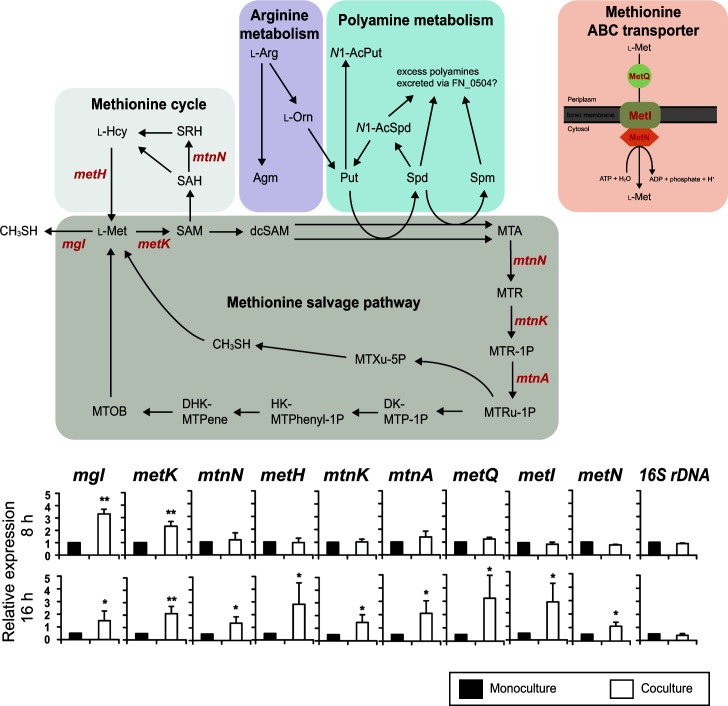
Relative fold changes in mRNA expression by *F. nucleatum* after coculture with *S. gordonii*. The level of mRNA expression for each incubation time was normalized to 16S rDNA of *F. nucleatum*. Fold changes were calculated using the following equation: fold = (mRNA expression by *F. nucleatum* in coculture with *S. gordonii*)/(mRNA expression by *F. nucleatum* in monoculture). Results are shown as the mean ± SD of three independent experiments. *P < 0.05, **P < 0.01 (two-tailed paired *t*-test, monoculture vs. coculture). Abbreviations are described in the legend in Fig. 5.

The one-carbon unit of MTA is known to be recycled to the methionine cycle via the methionine salvage pathway (45). Transcriptional changes in genes involved in the methionine cycle and methionine salvage pathway and also methionine ABC transporters were examined. After 8 and 16 h of incubation, the transcriptional levels of *mgl* and *metK* in cocultures of *F. nucleatum* and *S. gordonii* were increased by 2.3- to 4.2-fold, as compared to those in monocultures (Fig. 6). Although the expression levels of genes other than *mgl* and *metK* were not significantly changed after 8 h, upregulation of the expression of these metabolic genes by *F. nucleatum* was noted in cocultures after 16 h (Fig. 6). In particular, the expression level of *metK*, *metH*, *metQ*, and *metI* was markedly enhanced by 4.0- to 5.6-fold after a 16 h culture, indicating enhanced activities in methionine cycle and uptake and salvage pathway, especially under lower methionine concentration environments. Thus, enhanced methionine uptake likely occurs when cocultured with *S. gordonii*, as shown in Fig. 3, leading to increased CH_3_SH production. This is achieved through the upregulation of *mgl* and *metK* and activation of the methionine salvage pathway, involving MTA and MTRu-1P, and is also linked to polyamine synthesis.

These results suggest that ornithine secreted by *S. gordonii* promotes polyamine biosynthesis by *F. nucleatum*, resulting in a compensatory increase in demand for methionine, leading to elevated methionine metabolism and CH_3_SH production.

## DISCUSSION

For this investigation, a novel noncontact method for quantitation of CH_3_SH was developed (Fig. S1), which showed that nutritional cross-feeding enhances CH_3_SH production by *F. nucleatum* through altered methionine metabolism. The findings illustrate that metabolic interactions can modulate the emission of microbial volatile compounds, thus potentially contributing to the development of halitosis.

Consistent with several previous reports, the present study found that *F. nucleatum* is a potent producer of CH_3_SH. Interestingly, *F. nucleatum* released the highest amounts of CH_3_SH at pH 8.5 (Table 1; Fig. 1A), which was considered to be due to the optimal pH of METase (l-methionine + H_2_O → CH_3_SH + NH_3_ +2-oxobutanoate) of 8.0–8.5 in *F. nucleatum* as well as other bacteria (46–48). It has also been reported that an alkaline condition (pH 8.2) induces biofilm development by *F. nucleatum* through the increased abundance of adhesion proteins (49). The periodontal pocket in periodontitis patients has been shown to be alkaline, as high as pH 8.9 (49, 50). Hence, diseased periodontal pockets may harbor increased levels of *F. nucleatum*-related biofilms, from which larger amounts of CH_3_SH are emitted, thus contributing to malodor generation.

Although a number of studies have shown that interactions between oral bacteria elevate pathogenicity through enhancement of biofilm formation and increased adherence to and invasion of epithelial cells (27–30, 35–37), little is known regarding how these interactions affect oral malodor generation. In the present study, coculture of *S. gordonii* with *F. nucleatum* facilitated CH_3_SH production, particularly when supplemented with methionine at concentrations similar to those seen in a natural oral environment. Interestingly, *A. naeslundii* suppressed CH_3_SH production by *F. nucleatum* and also nearly abolished its production by other periodontal pathogens including *P. gingivalis* (Fig. 1C). These findings are consistent with those observed in studies of soil bacterial communities, where interspecies interactions have been found to either promote or constrain volatile production (51). It is therefore likely that oral polymicrobial communities exhibit enhanced or suppressed CH_3_SH production depending on interactions between the community members, which highlights the need for studies to resolve individual roles of different species.

The noncontact coculture experiments showed that the exchange of diffusible molecules from *S. gordonii* enhances the level of CH_3_SH generation by *F. nucleatum*. Additional examination also revealed that this interaction is driven by the metabolism of ArcD-excreted ornithine by the ODC of *F. nucleatum*. These findings add to a growing body of evidence showing the importance of microbial metabolic interactions that integrate microbial communities and affect the pathogenicity of oral diseases (52). In particular, our recent study showed the cooccurrence of *P. gingivalis* with the genes of *S. gordonii arcD* and *F. nucleatum* ODC in periodontitis patients. It demonstrated that ornithine cross-feeding via ArcD of *S. gordonii*-induced ODC-catalyzed polyamine production by *F. nucleatum*, thus enhancing the biofilm lifecycle of *P. gingivalis* (53). Therefore, the present findings highlight the importance of engagement of *F. nucleatum* in a cross-feeding network with *S. gordonii*, not just with regard to periodontal pathogenesis but also disease-associated halitosis.

Notably, we found that ornithine cross-feeding promotes the uptake and metabolism of methionine by *F. nucleatum*. Methionine is an important molecule for the initiation of protein synthesis and SAM-mediated methylation of proteins, RNA, and DNA (54–56). Metabolic routes of methionine incorporated in *F. nucleatum* can be divided mainly into methionine cycle and salvage pathways (45, 57, 58). The former pathway functions to recycle adenine and methionine through a SAM-mediated methylation reaction, leading to the production of AI-2, a QS signal (59, 60). *F. nucleatum* AI-2 has been shown to have an important role in inter- and intraspecies interactions in microbial communities, thus affecting periodontal pathogenesis (61). Hence, it cannot be ruled out that an elevated level of AI-2 molecules in *F. nucleatum* can result in greater levels of CH_3_SH production. To examine that possibility, the enhancement of CH_3_SH production was assessed by the addition of DPD, an AI-2 precursor. The results showed that DPD/AI-2 had no significant effect on CH_3_SH production, indicating that an AI-2-based QS system is not involved in increased CH_3_SH production. On the other hand, labeling experiments indicated a slight increase in the ratio of methionine (m + 5) over time, suggesting that a portion of labeled methionine (m + 6) enters the methionine cycle for methylation, as well as resynthesis and reuse of methionine.

The methionine salvage pathway is known to be involved in various cellular processes, including the preservation of intracellular sulfur pools for the formation of amino acids and proteins, and also the synthesis of polyamines, such as putrescine, spermine, and spermidine, which are important molecules for cell growth, biofilm formation, and protection from oxidative and acid stress (62–64). Results from labeling experiments with ^13^C, ^15^N-methionine showed a high similarity of labeling between SAM (m + 6) and MTA (m + 1), demonstrating that labeled methionine enters the polyamine pathway (Fig. 5A). A longer incubation period (6 h) resulted in gradual increases in SAM (m + 7, m + 8) and MTA (m + 2, m + 3) (Fig. 5B), indicating possible production of SAM (m + 7, m + 8) from methionine (m + 6) and labeled ATP (m + 1, m + 2), the latter of which was synthesized from labeled methionine via a *de novo* ATP synthesis pathway. The purine carbon skeleton is composed of two nitrogens from Gln, two from Asp and Gly each, one carbon from *N*^10^-formyl-THF, and one from *N*^5^*N*^10^-methenyl-THF. As shown in Fig S6, METase metabolizes l-methionine and produces NH_3_ (m + 1), CH_3_SH (m + 1), and 2-oxobutyrate (2OB; m + 4) that leads to the one-carbon pool via formate (m + 1). As shown in Fig. S7, *F*. *nucleatum* produces *N*^10^-formyl-THF (m + 1) and *N^5^N^10^*-methenyl-THF (m + 1) in the one-carbon pool. Therefore, it seems natural that SAM (m + 7 and m + 8) and MTA (m + 2, m + 3) would be present after a certain time. On the other hand, despite findings showing methionine-derived labeling in MTA (m + 1), no labeling in spermidine (m + 4) was detected. This may be explained by the function of FN_0504, a putative L-ornithine/polyamine antiporter, to efflux polyamines as it takes up L-ornithine. In addition, the adsorption of spermidine onto capillary walls causes peak broadening, resulting in reduced detection limits. Results from UPLC and labeling experiments showed that *S. gordonii* secreted ornithine, which led to a dramatic increase in intracellular ornithine and polyamines, indicating an increase in polyamine pathway activity (Fig. 3 and 5A), a finding consistent with our previous report (53). Additionally, acetylated polyamines were detected in *F. nucleatum* cells, although that was dependent on the levels of putrescine and spermidine (Fig. 5A), suggesting that excess levels of these may cause their acetylation and maintain intracellular levels of polyamines at a constant level. Acetylation of excess polyamines by diamine *N*-acetyltransferase [FN_1057; EC 2.3.1.57] in *F. nucleatum* requires acetyl-CoA. *In silico* analysis of acetyl-CoA biosynthetic pathways from L-methionine revealed only one pathway for incorporating a ^13^C into acetylputrescine (Fig S7). Notably, *F. nucleatum* cannot complete this pathway alone; it requires the enzymatic reaction of glycine hydroxymethyltransferase [SGO_1151; EC 2.1.2.1], encoded by *S. gordonii* (Fig S7). We previously confirmed that *S. gordonii* releases serine into the environment (unpublished data), and *F. nucleatum* has been reported to take up and utilize serine (65). Therefore, serine-mediated crossfeeding between the two species should be possible. Anaerobically grown *S. gordonii* has been shown to be able to produce H_2_O_2_ when glucose is available, albeit to a lesser extent than under an aerobic condition (66), thus *F. nucleatum* might increase the elevation of intracellular polyamines in response to H_2_O_2_ generated by *S. gordonii*. It is also considered likely that the elevated polyamine-synthesis pathway activity under coexistence with *S. gordonii* increases the demand for methionine, following enhancement of methionine metabolism and CH_3_SH generation.

Considering that the ratio of methionine (m + 1) showed a slight increase over time (Fig. 5B), a portion of accumulated MTA was likely resynthesized to methionine (m + 1) via 4-methylthio-2-oxobutanoic acid (MTOB). Although biosynthesis of MTOB from *S*-methyl-5-thio-d-ribose 1-phosphate (MTRu-1P) reportedly requires oxygen (67, 68), as also noted in the present experiments (Fig. 6), recent studies by North et al. show that *Rhodospirillum rubrum* possesses an oxygen-independent MTA-isoprenoid shunt that links MTA metabolism to the release of CH_3_SH for methionine regeneration and 1-deoxyxylulose-5-phosphate (DXP) synthesis for isoprenoid metabolism under anaerobic conditions (Fig. 6) (67–69). *F. nucleatum* may produce CH_3_SH via an MTA-isoprenoid shunt under anaerobic coculture conditions, although further study is required to determine the precise MTA-isoprenoid shunt in *F. nucleatum*. l-Methionine regeneration might also occur through FN_1745, a cystathionine gamma-synthase [EC 2.5.1.48]. This enzyme typically facilitates cystathionine production from *O*-succinyl-l-homoserine and l-cysteine. It is reportedly capable of producing l-methionine when CH_3_SH is used instead of l-cysteine (70). However, its *K_m_* value with CH_3_SH is significantly higher than with l-cysteine, and the *V_max_* is notably low (70), suggesting that this reaction is a minor, if not negligible, pathway for l-methionine regeneration.

The sustainable resynthesis of l-methionine through three distinct pathways, the methionine cycle, methionine salvage, and the activity of FN_1745, likely contributes to the continuous release of CH_3_SH in cocultures of *F. nucleatum* with *S. gordonii* compared to *F. nucleatum* monocultures. *F. nucleatum* has three closely related species previously classified as subspecies: *Fusobacterium polymorphum*, *Fusobacterium vincentii*, and *Fusobacterium animalis. F. polyrmorphum* and *F. vincentii* also possess genes related to CH_3_SH production, including *mgl*, *metK,* and *metQ*. This indicates the possibility that the coexistence of these bacteria and *S. gordonii* enhances CH_3_SH production. Thus, our results suggest that this phenomenon could potentially occur in coexistence with various bacteria that possess related genes.

**Fig 7:**
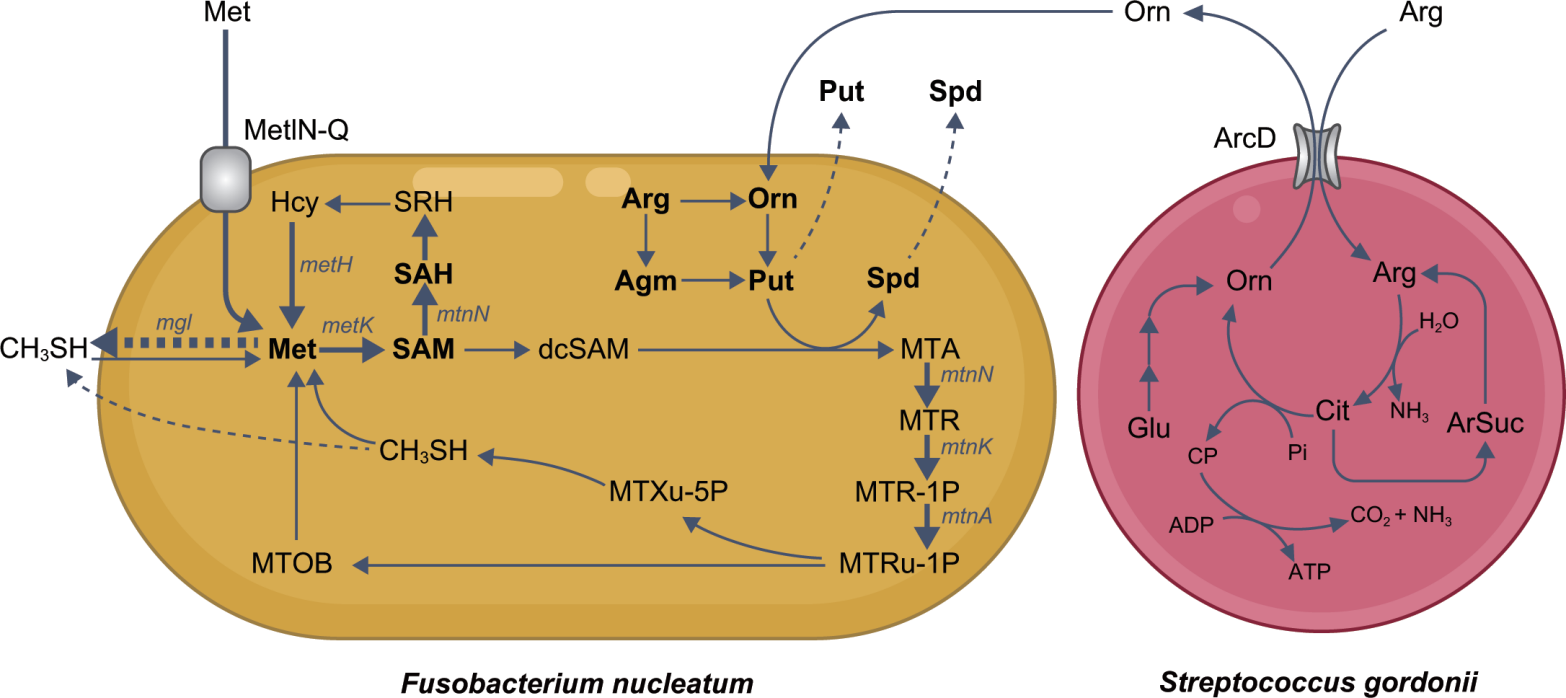
Schematic representation of the observed metabolic flow of bacterial metabolism in *F. nucleatum* and *S. gordonii* cocultures. *S. gordonii* takes up l-arginine and excretes ornithine extracellularly. *F. nucleatum* activates ornithine metabolism and synthesizes polyamines via the methionine salvage pathway, after which the uptake of extracellular methionine is accelerated, and metabolic flow is shunted to the MTA synthesis pathway. Moreover, methionine is resynthesized via the methionine cycle and potentially via FN_1745. Detected metabolites are shown in bold, with dashed arrows for excretion and bold arrows for confirmed upregulation of bacterial metabolism. Cit, citrulline; Glu, glutamate; ArSuc, arginosuccinate; Pi, inorganic phosphate; CP, carbamoyl phosphate; others detailed in the Fig. 5 legend.

The current study confirmed that *S. gordonii* takes up extracellular arginine via ArcD and produces ornithine intracellularly. The metabolism of ornithine excreted by *S. gordonii* leads to enhanced uptake and regeneration of methionine in *F. nucleatum*, driven by increased polyamine synthesis, thereby boosting CH_3_SH production (Fig. 7). Although the number of species used was limited, new insights regarding the impact of metabolic cross-feeding in microbial communities on the generation of malodor compounds in the oral cavity were obtained. Nevertheless, a wide range of metabolites are exchanged among oral bacteria. Thus, further work is needed to fully understand the implications of microbial metabolic interactions related to the development of halitosis.

## Data Availability

This study is available at the NIH Common Fund’s National Metabolomics Data Repository (NMDR) website, the Metabolomics Workbench, https://www.metabolomicsworkbench.org, where it has been assigned Study ID ST002793. The data can be accessed directly via its Project DOI: http://dx.doi.org/10.21228/M8P126.

## SUPPLEMENTAL MATERIAL

Data Set S1 msystems.00764-23-s0001.xlsx Data from methionine stable isotope experiment.

Supplemental Figures msystems.00764-23-s0002.docx Fig. S1, S2, S3, S4, S5, S6, and S7.

Additional details msystems.00764-23-s0003.docx Supplement to the experimental procedure and an additional list of references.

Supplemental Tables msystems.00764-23-s0004.docx Tables S1 and S2.

ASM does not own the copyrights to Supplemental Material that may be linked to, or accessed through, an article. The authors have granted ASM a non-exclusive, world-wide license to publish the Supplemental Material files. Please contact the corresponding author directly for reuse.
