# Supplementary material for: Interspecies metabolite transfer fuels the methionine metabolism of Fusobacterium nucleatum to stimulate volatile methyl mercaptan production
Source: mSystems. 2024 Jan 30;9(2):e00764-23. doi: 10.1128/msystems.00764-23 (PMC10878106; doi:10.1128/msystems.00764-23)
Supplement: Supplemental Figures — Fig. S1, S2, S3, S4, S5, S6, and S7. [file msystems.00764-23-s0002.docx]

**SUPPLEMENTAL FIGURES**

**
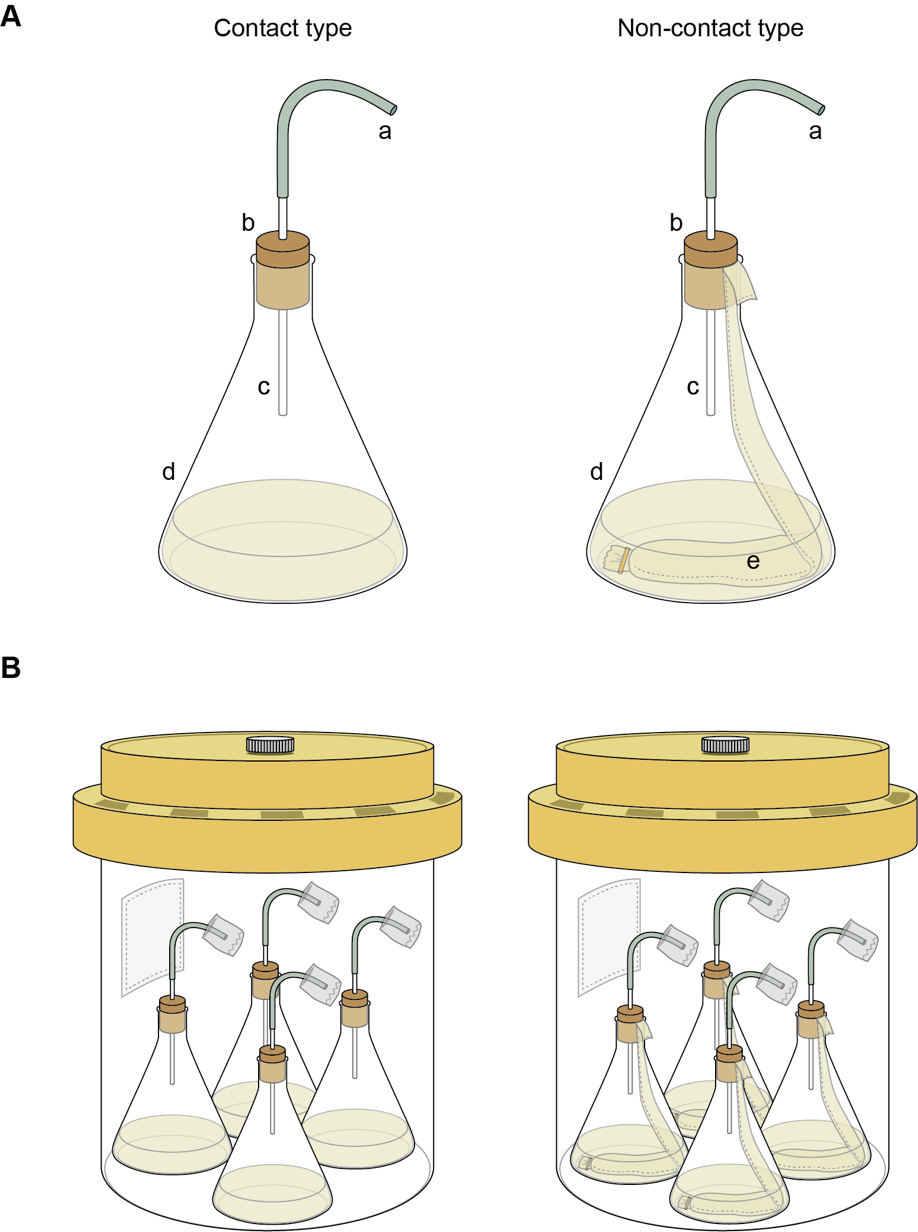
**

**Fig. S1. Anaerobic/Microaerophilic and non-contact culture systems.**

A. Individual culture setup. a, silicone tube; b, rubber stopper; c, stainless tube; d, 200 mL Erlenmeyer flask; e, dialysis tube. Bacterial cultures in flasks were anaerobically incubated at 37°C using a jar (The GasPak 150 jar, Becton, Dickinson and Company, US). Silicon tubes (a) were equipped to create anaerobic or microaerophilic conditions and minimize the emission of volatile gases in the flasks.
B. Group culture incubation system. A set of four flasks of bacterial cultures for each experimental group were incubated in a jar (The GasPak 150 jar, Becton, Dickinson and Company, NJ, US) at 37°C either anaerobically or microaerobically to minimize contamination by volatile compounds emitted from different experimental groups.

**
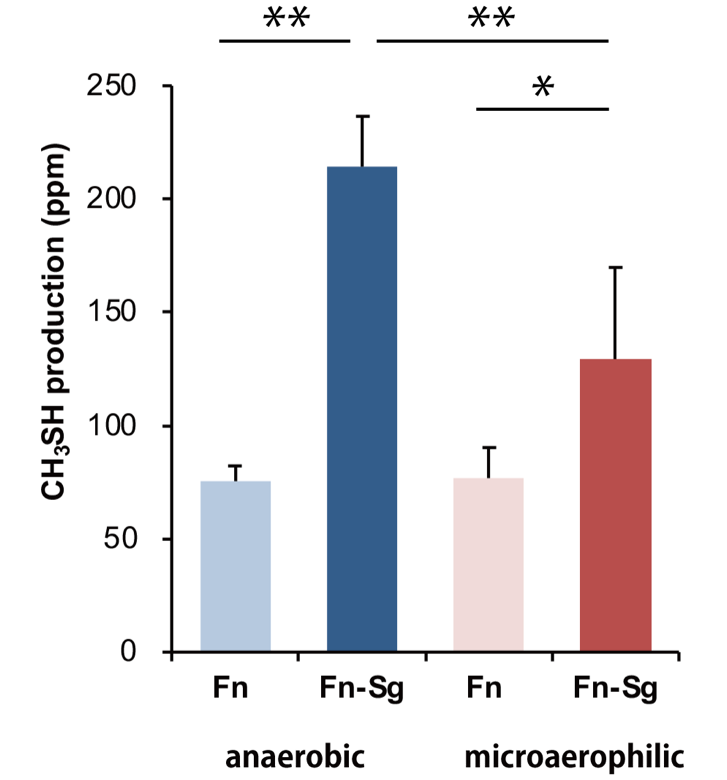
**

**Fig. S2. Comparison of CH_3_SH production capacity under anaerobic and microaerophilic conditions.**

Bacterial cultures were supplemented with 1.0 mM of l-methionine. Each group was incubated separately for 16 hours in a jar system under anaerobic or microaerophilic conditions. Microaerophilic conditions were prepared using AnaeroPack^TM^-MicroAero (Mitsubishi Gas Chemical, Tokyo, Japan). Results are shown as the mean ± SD of four independent experiments. **p* <0.05, ***p* <0.01 (one-way ANOVA, followed by Scheffe test)


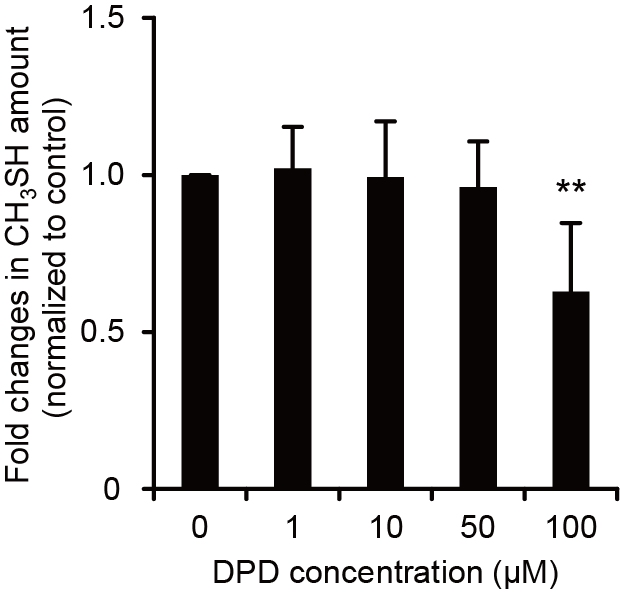


**Fig. S3. Fold changes in CH_3_SH level by addition of various concentrations of DPD to *F. nucleatum* monocultures.**

The fold level was normalized to that of the control sample without DPD. *F. nucleatum* cultures supplemented with 1.0 mM methionine were anaerobically incubated for 16 h. Results are shown as the mean ± SD of four independent experiments. ***p* <0.01 (one-way ANOVA, followed by Dunnett's test).

**
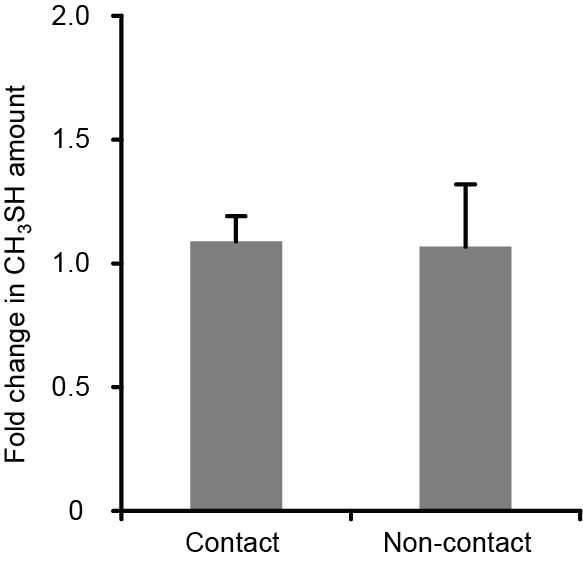
**

**Fig. S4. Change of CH_3_SH production in cocultures of *F. nucleatum* with Δ*arcD* in contact and non-contact culture systems.**

Bacterial cultures were supplemented with 1.0 mM of l-methionine. Results are shown as the mean ± SD of four independent experiments. Fold changes were calculated using the following equation: fold = (amount of CH_3_SH formation in coculture) / [(amount of CH_3_SH formation in single culture of *F. nucleatum*) + (amount of CH_3_SH formation in single culture of Δ*arcD*)]. All cultures were anaerobically incubated for 16 h, as described in materials and methods.

**
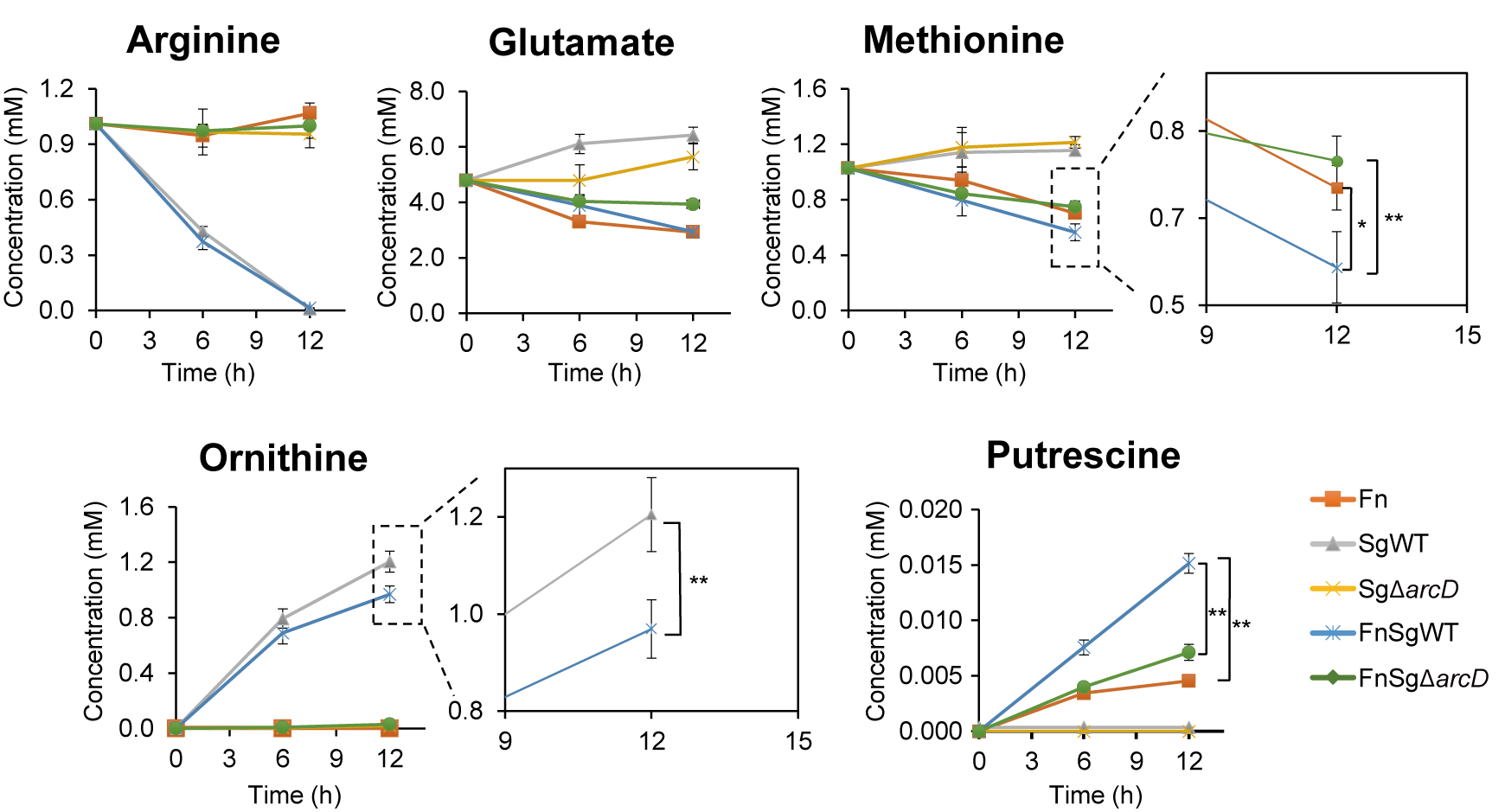
**

**Fig S5. Time course of changes in extracellular metabolites in culture fluids.**

Lines indicate *F. nucleatum* (orange), *S. gordonii* WT (gray), and *S. gordonii* ΔarcD (yellow) monocultures, and *F. nucleatum* and *S. gordonii* WT (blue), and *F. nucleatum* and *S. gordonii* ΔarcD (green) cocultures. Results are shown as the mean ± SD of three independent experiments. **p* <0.05, ***p* <0.01 (one-way ANOVA, followed by Tukey–Kramer post-hoc test) for methionine and ornithine metabolism. For clarity, some of the data in Figure 3 are repeatedly shown.


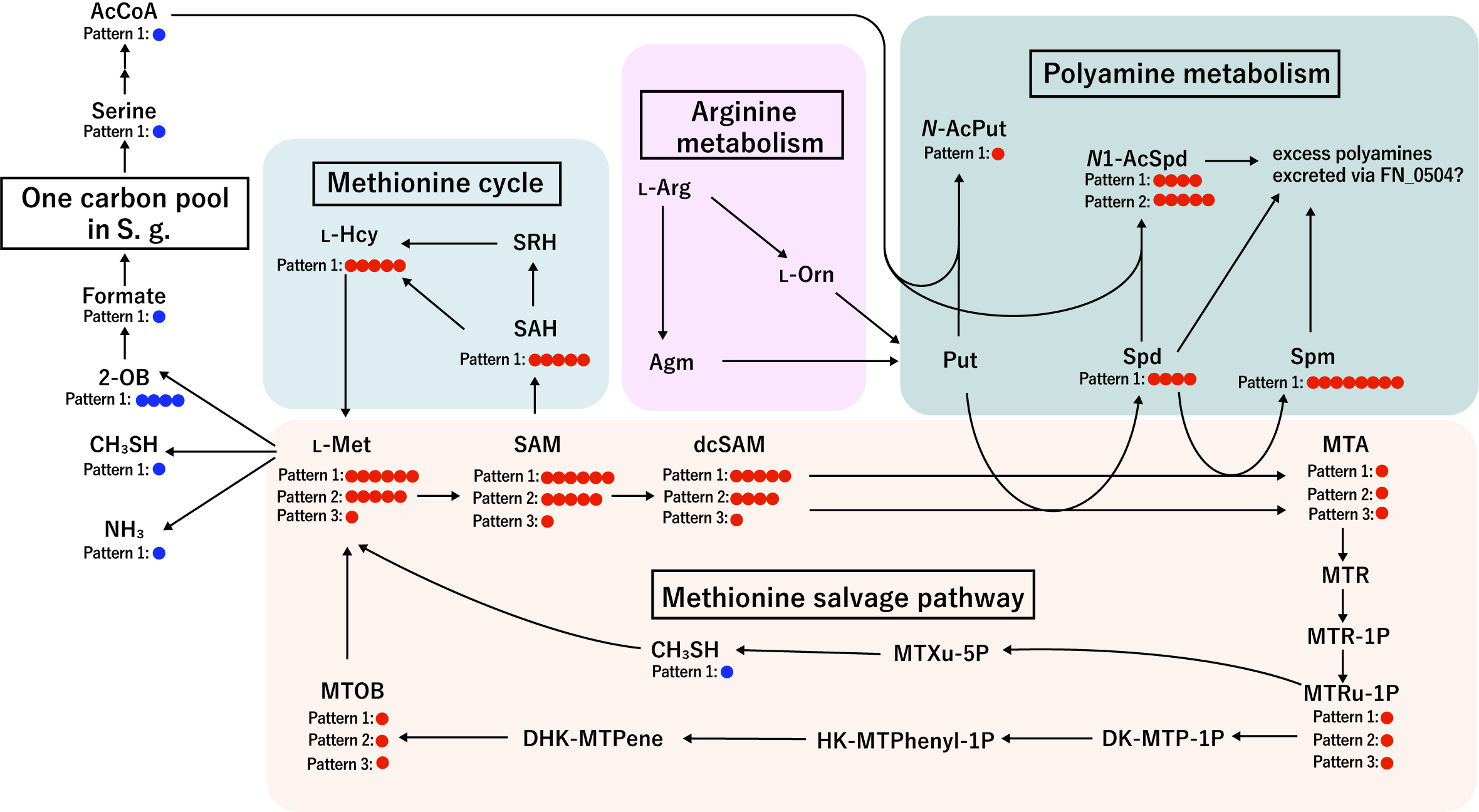


**Fig. S6. Predicted flux profiling of methionine metabolic pathways in *F. nucleatum*.** Shown are expected ^13^C, ^15^N-labelling patterns in methionine cycle, and methionine salvage pathway intermediates and byproducts with [^13^C_5_, ^15^N] l-methionine used as tracer. Red circle shows number of labelling atoms in targeted metabolites. Blue circles indicate the estimated number of labeled atoms for metabolites not measured in this study. As shown in Figure S7, there is a metabolic reaction that proceed only by *S. gordonii* in one carbon pool.

Abbreviations: Met, l-methionine; SAM, S-adenosyl- l-methionine; SAH, *S*-adenosyl- l-homocysteine; SRH, *S*-ribosyl-l-homocysteine; l-Hcy, l-homocysteine; dcSAM, *S*-adenosylmethioninamine; MTA, 5'-methylthioadenosine; l-Arg, l-arginine; Agm, agmatine; l-Orn; l-ornithine; Put, putrescine; *N*-AcPut, *N*-acetylputrescine; *N*1-AcSpd, *N*1-acetylspermidine; Spd, spermidine; Spm, spermine; MTR, 5-methylthio-d-ribose; MTR-1P, *S*-methyl-5-thio-d-ribose 1-phosphate; MTRu-1P, *S*-Methyl-5-thio-d-ribulose 1-phosphate; MTXu-5P, 1-(methylthio)xylulose 5-phosphate; CH_3_SH, methyl mercaptan; DK-MTP-1P, 2,3-diketo-5-methyl-thiopentyl-1-phosphate; HK-MTPhenyl-1P, 2-hydroxy-3-keto-5-methylthiopentenyl-1-phosphate; DHK-MTPene, 1,2-dihydroxy-5-(methylthio) Pent-1-en-3-one; MTOB, 4-methylthio-2-oxobutanoic acid; 2-OB, 2-oxobutanoate; AcCoA, acetyl-coenzymeA.

**
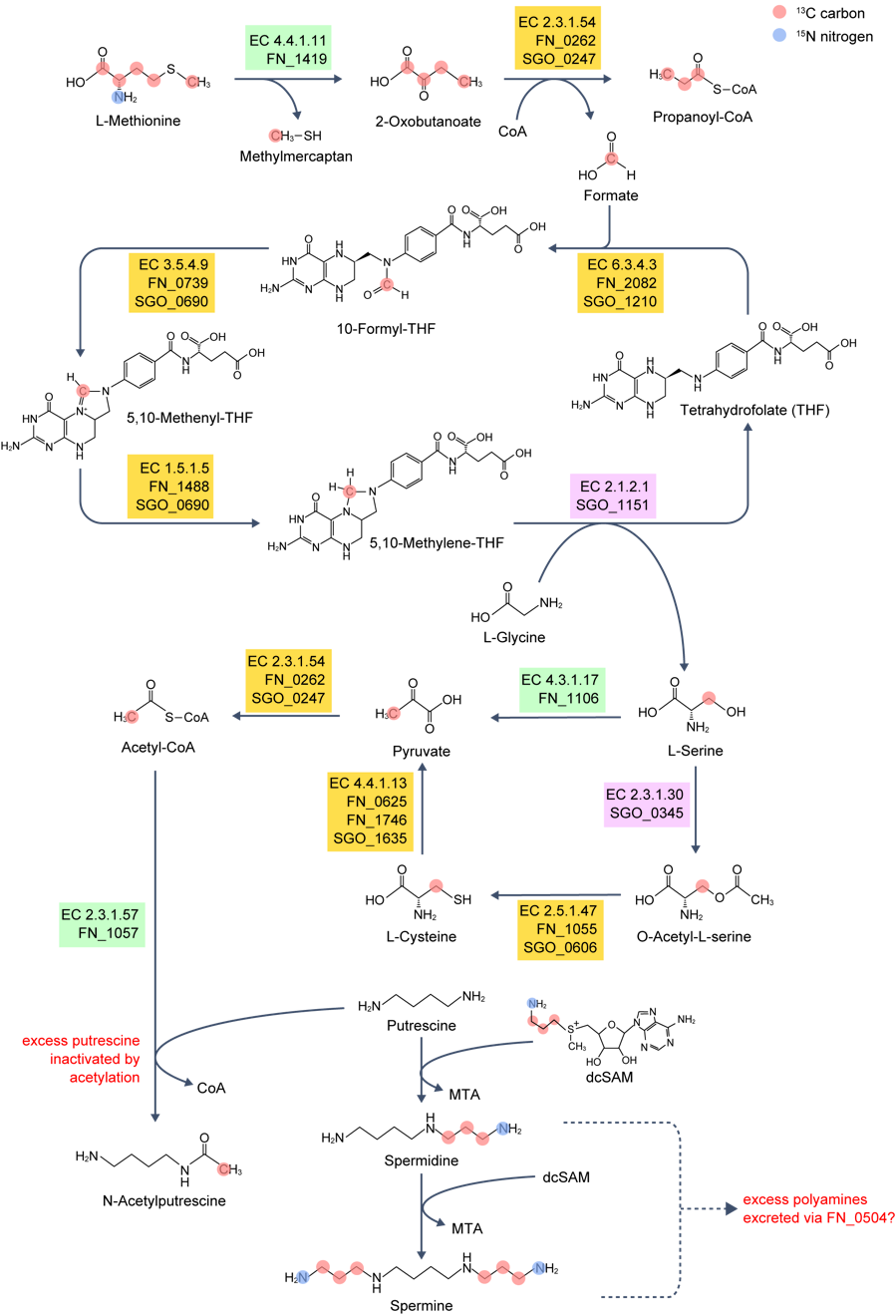
**

**Fig. S7. Putative flux profiling of a methionine metabolic pathway producing *N*-acetyl-putrescine with a ^13^C.**

Shown are expected ^13^C, ^15^N-labelling patterns in a methionine metabolic pathway to produce m+1 *N*-acetyl-putrescine. Red and blue circles indicate ^13^C and ^15^N, respectively. Note that some metabolic reactions proceed only by *S. gordonii* or *F. nucleatum*. Enzyme reactions with green background color indicate reactions annotated only with *F. nucleatum*, those with pink background color indicate reactions annotated only with *S. gordonii*, and those with yellow background color indicate reactions annotated with both *F. nucleatum* and *S. gordonii*.
