# Supplementary material for: Interspecies metabolite transfer fuels the methionine metabolism of Fusobacterium nucleatum to stimulate volatile methyl mercaptan production
Source: mSystems. 2024 Jan 30;9(2):e00764-23. doi: 10.1128/msystems.00764-23 (PMC10878106; doi:10.1128/msystems.00764-23)
Supplement: Additional details — Supplement to the experimental procedure and an additional list of references. [file msystems.00764-23-s0003.docx]

**SUPPLEMENTAL INFORMATION**

**EXTENDED EXPERIMENTAL PROCEDURES**

**UPLC condition**

Liquid chromatographic separation was performed using an AccQ•TagTM Ultra column (2.1 i.d. × 100 mm, 1.7-μm particle size, Waters, USA) at 43°C with a flow rate of 0.7 mL/min. Mobile phases A-D: A = 100% Waters AccQ-Tag Eluent A; B = 10% Waters AccQ-Tag Eluent B; C = 100% Milli-Q water; D = 100% Waters AccQ-Tag Eluent B. The gradient was as follows: T = 0, 9.9% A, 90.1% C; T = 0.29 min, 9.9% A, 90.1% C;T = 4.84 min, 9.1% A, 70% B, 20.9% C, 0% D; T = 6.45 min, 8% A, 15.6% B, 58.9% C, 17.5% D; T = 6.65 min, 8% A, 15.6% B, 58.9% C, 17.5% D; T = 7.04 min, 7.8% A, 0% B, 71.9% C, 20.3% D; T = 7.64 min, 13.7% A, 0% B, 36.3% C, 50% D ; T = 8.89 min, 13.7% A, 0% B, 36.3% C, 50% D ; T = 8.98 min, 9.9%A, 0% B, 90.1% C, 0% D. Signals were detected at a wavelength of 260 nm. One microliter of the sample and standards were injected for analysis. Concentrations of amino acids were calculated using an external standard calibration curve. Data were analyzed using the Empower 2 software package.

**Intracellular metabolite analysis by CE-TOFMS**

To investigate the time course of changes in intracellular metabolites of *F. nucleatum* cells after coculturing with *S. gordonii* WT, *F. nucleatum* cells were collected by centrifugation (7670 × *g* for 7 min at 4°C), washed twice with 1 ml of ultra-pure water, and then immediately treated twice with 1 ml of 100% methanol containing the internal standards (H3304-1002, Human Metabolome Technologies, Inc., Tsuruoka, Japan). The extract was then centrifuged at 2300 × *g* and 4ºC for 5 min, after which 2 mL of the upper aqueous layer was centrifugally filtered through a Millipore 5-kDa cutoff filter at 9100 × *g* and 4ºC for 120 min to remove proteins. Subsequently, the filtrate was centrifugally concentrated and re-suspended in 50 µL of Milli-Q water for CE-MS analysis. Measurements of extracted intracellular metabolites and supernatants after incubation in both positive and negative modes were done using CE-TOFMS by Human Metabolome Technologies (HMT; Tsuruoka, Japan). with an Agilent CE Capillary Electrophoresis System equipped with an Agilent 6210 Time of Flight mass spectrometer, Agilent 1100 isocratic HPLC pump, Agilent G1603A CE-MS adapter kit, and Agilent G1607A CE-ESI-MS sprayer kit (Agilent Technologies, Waldbronn, Germany). The systems were controlled by the Agilent G2201AA ChemStation software package, version B.03.01, for CE (Agilent Technologies, Waldbronn, Germany). Metabolites were analyzed using a fused silica capillary (50

μm *i.d.*

× 80

cm total length), with commercial electrophoresis buffer (Solution ID: H3301-1001 for cation analysis, H3302-1021 for anion analysis, Human Metabolome Technologies) as the electrolyte. The sample was injected at a pressure of 50

mbar for 10 s (approximately 10

nL) for cation analysis and 25

s (approximately 25

nL) for anion analysis. The spectrometer was scanned from 50 to 1000 m/z. Other related conditions have been described in previous reports (1–3). The relative amount of each metabolite was calculated with reference to the HMT internal standard materials. Metabolite concentrations were normalized to viable cell counts. The concentration of each of the isotopomers was calculated based on the natural abundance ratio of the stable isotope.

**DPD/AI-2 supplementation**

4, 5-Dihydroxy-2, 3-pentanedione (DPD, Carbosynth Limited), referred to as DPD/AI-2 because of its spontaneous conversion into AI-2, was used as a supplement for enhancement of CH_3_SH generation by *F. nucleatum*. DPD was added at a final concentration of 1 to 100 μM to mCDM solution containing 1.0 mM l-methionine. The culture protocol method and quantitation of CH_3_SH level were performed as described above.

**REFERENCES**

1. Soga T, Heiger DN. Amino acid analysis by capillary electrophoresis electrospray ionization mass spectrometry. *Anal Chem* 2000; **72**: 1236–1241.

2. Soga T, Ueno Y, Naraoka H, Ohashi Y, Tomita M. Nishioka T. Simultaneous determination of anionic intermediates for *Bacillus subtilis* metabolic pathways by capillary electrophoresis electrospray ionization mass spectrometry. *Anal Chem* 2002; **74**: 2233–2239.

3. Soga T, Ohashi Y, Ueno Y, Naraoka H, Tomita M. Nishioka T. Quantitative metabolome analysis using capillary electrophoresis mass spectrometry. *J Proteome Res* 2003; **2**: 488–494.
