# Supplementary material for: Interspecies metabolite transfer fuels the methionine metabolism of Fusobacterium nucleatum to stimulate volatile methyl mercaptan production
Source: mSystems. 2024 Jan 30;9(2):e00764-23. doi: 10.1128/msystems.00764-23 (PMC10878106; doi:10.1128/msystems.00764-23)
Supplement: Supplemental Tables — Tables S1 and S2. [file msystems.00764-23-s0004.docx]

**SUPPLEMENTAL TABLES**

**Table S1. Oligonucleotide sequences and gene targets of primers used for real-time PCR amplification**

| Gene target | Oligonucleotide sequence (5’ → 3’) | Reference |
| --- | --- | --- |
| *mgl* | F; ACAGGAGCTGTTTTAGGACCTC  R; AACCAGGATGAGTCTCAAGTCC | Present study |
| *metK* | F; ATTGGAGGAGCAGGAGATCAAG  R; TGATCTGGTCTTGCCCATTC | Present study |
| *mtnN* | F; TGGCAGAATCAGTAGCAAGC  R; TCCATCTCAACGCATTCAGC | Present study |
| *metH* | F; TCAGTAGGACCAACAAATAAGGG  R; CCACCATCTATAAGTCCTGCTAC | Present study |
| *mtnK* | F; ACTTCGTTCATCAGGAAGACC  R; TCCTGCTATAAGCTCTGTTCTC | Present study |
| *mtnA* | F; TGTAGGTTGTATGGGGAGAGAG  R; TGTCCAAGCGACCATATTATCTG | Present study |
| *metQ* | F; AGTTGGAGCAACACCAGTTC  R; GGTTTGTGTTGGAAGAAGTTAGC | Present study |
| *metI* | F; TCAACAATAGGGGCAACAGC  R; TGCTAGCTCCCATACTTTGAC | Present study |
| *metN* | F; TCAGCACTTGATCCTAAAACAAC  R; TGTACACCACCTTGTTCGAC | Present study |
| 16S rRNA | F; TGCAAGTCTACTTGAATTTGGGTTT  R; CTAGCTGTGAGGCAAGTTCTTTAC | Appl Environ Microbiol. 75, 7725–7733 (2009) |

**Table S2. Changes in optical density (ΔOD) of bacterial culture in mCDM before and after incubation.**

|  | **ΔOD (16h – 0h)** | |
| --- | --- | --- |
| **Bacterial strain** | **mean** | **SD** |
| ***Streptococcus gordonii* DL1** | **-0.07** | **0.01** |
| ***Actinomyces naeslundii* ATCC19039** | **0.67** | **0.02** |
| ***Fusobacterium nucleatum* subsp. *nucleatum* ATCC25586** | **-0.07** | **0.02** |
| ***Prevotella intermedia* ATCC49046** | **-0.02** | **0.01** |
| ***Filifactor alocis* ATCC35846** | **-0.04** | **0.01** |
| ***Porphylomonas gingivalis* ATCC33277** | **-0.10** | **0.01** |
